# Sialic acid-binding lectin (leczyme) induces apoptosis to malignant mesothelioma and exerts synergistic antitumor effects with TRAIL

**DOI:** 10.3892/ijo.2013.2192

**Published:** 2013-11-28

**Authors:** TAKEO TATSUTA, MASAHIRO HOSONO, KOHTA TAKAHASHI, TAKASHI OMOTO, YUKIKO KARIYA, SHIGEKI SUGAWARA, SENITIROH HAKOMORI, KAZUO NITTA

**Affiliations:** 1 Division of Cell Recognition Study, Institute of Molecular Biomembrane and Glycobiology, Tohoku Pharmaceutical University, Aoba-ku, Sendai 981-8558;; 2 Fukushima Medical University, Fukushima 960-1295, Japan;; 3 Division of Biomembrane Research, Pacific Northwest Research Institute, WA 98122, USA

**Keywords:** lectin, ribonuclease, leczyme, malignant mesothelioma, TRAIL, Bid, synergistic antitumor effect

## Abstract

Malignant mesothelioma is a highly aggressive tumor with poor prognosis. An effective drug for treatment of malignant mesothelioma is greatly needed. Sialic acid-binding lectin (SBL) isolated from oocytes of *Rana catesbeiana* is a multifunctional protein which has lectin activity, ribonuclease activity and antitumor activity, so it could be developed as a new type of anticancer drug. The validity of SBL for treatment of malignant mesothelioma was assessed using three malignant mesotheliomas and a non-malignant mesothlial cell line. Effectiveness of combinatorial treatment of SBL and tumor necrosis factor-related apoptosis inducing ligand (TRAIL) was also elucidated and characterized. SBL induced tumor-selective cytotoxicity that was attributed to induction of apoptosis. Combinatorial treatment of SBL and TRAIL showed synergistic apoptosis-inducing effect. Additional experiments revealed that Bid was the mediating molecule for the synergistic effect in SBL and TRAIL. These results suggested that SBL could be a promising candidate for the therapeutics for malignant mesothelioma. Furthermore, the combinatorial treatment of SBL and TRAIL could be an effective regimen against malignant mesothelioma.

## Introduction

Malignant mesothelioma is a highly aggressive tumor with poor prognosis (1). It is commonly accepted that the development of malignant mesothelioma is closely associated with exposure to asbestos, radiation or simian virus 40 (2). Because of the fact that asbestos has been used in thousands of products for a long time all over the world, and estimated latency period between exposure to asbestos and diagnosis of mesothelioma is 20–40 years, and it is expected that the incidence of malignant mesothelioma increases dramatically over the next couple of decades (3,4). Malignant mesothelioma shows strong resistance to existing chemotherapy, irradiation therapy, and operative therapy and extremely poor prognosis with median survival and 5-year survival of <1 year and 1%, respectively (5,6). Because the benefits of single-agent first-line or second-line chemotherapy are limited, the current standard of care for first-line chemotherapy is cisplatin and pemetrexed (a folate inhibitor), the only first-line therapy approved by the US Food and Drug Administration for patients ineligible for surgery (7).

Sialic acid binding lectin (SBL) isolated from *Rana catesbeiana* is a multifunctional protein that has lectin activity, ribonuclease activity and antitumor activity. SBL selectively agglutinates tumor cells but not normal cells (8–10). The selective effect of SBL on cancer cells is due to its selective binding to tumor cells, because sialidase treatment of cells abolished the tumor cell agglutination and anti-proliferative effect induced by SBL (11). We previously reported the antitumor effect of SBL *in vitro* and *in vivo* (11–14), and the mechanism of SBL-inuduced apoptois was studied in human leukemia Jurkat cells (15,16). We studied the efficiency of SBL on treatment of malignant mesothelioma. We showed that SBL suppressed the cell proliferation of malignant mesothelioma and exerted synergistic apoptotic effect with tumor necrosis factor-related apoptosis inducing ligand (TRAL). The synergistic mechanism was analyzed and the potential of SBL as a new, active, anticancer reagent is suggested.

## Materials and methods

### Materials

SBL was isolated in sequential chromatography on Sephadex G-75, DEAE-cellulose, hydroxyapatite, and SP-Sepharose as described previously (17). Etoposide and anti-β-actin antibody were purchased from Sigma-Aldrich, (Tokyo, Japan). TRAIL was purchased from R&D Systems (Minneapolis, MN, USA). The antibodies utilized were: anti-caspase-9 (MBL, Nagoya, Japan), anti-caspase-8, anti-caspase-3, anti-Bim, anti-Bik, anti-Bax and anti-Bid (Cell Signaling Technology, Beverly, MA, USA), anti-GAPDH (Ambion, Austin, TX, USA), anti-ERK1/2 (pT202/pY204), anti-ERK1, anti-JNK/SAPK (pT183/pY185), anti-JNK/SAPK, anti-p38 (pT180/pY182), and anti-p38 (BD Biosciences, Franklin Lakes, NJ, USA), horseradish peroxidase (HRP)-conjugated anti-mouse IgG (Zymed, South San Francisco, CA, USA), and HRP-conjugated anti-rabbit IgG (Cedarlane, Hornby, Ontario, Canada). Bid specific siRNA were obtained from Ambion.

### Cell culture

Malignant mesothelioma cell line NCI-H28 and immortalized non-malignant mesothelial cell line Met-5A were purchased from American Type Culture Collection (ATCC; Manassas, VA, USA). Malignant mesothelioma cell lines ACC-MESO-1 and ACC-MESO-4 were obtained from Riken Cell Bank (Tsukuba, Japan). H28, MESO-1 and MESO-4 cells were cultured in RPMI-1640 medium supplemented with 10% fetal bovine serum (FBS). Met-5A was cultured in Medium 199 with Earle's balanced salt solution, 75 mM L-Gln, and 1.25 g/l sodium bicarbonate, supplemented with 3.3 nM epidermal growth factor (EGF), 400 nM hydrocortisone, 870 nM insulin, 20 mM HEPES, and 10% FBS. All cells were cultured with penicillin (100 U/ml) and streptomycin (100 *μ*g/ ml) at 37°C in 95% air and 5% CO_2_ atmosphere.

### Clonogenic assay

Cells were precultured for 24 h, and treated with SBL at various concentration for 48 h. Thereafter, the cells were trypsinized, and washed with phosphate-buffered saline (PBS), and plated in a 6-well plate (MESO-1, 500; MESO-4 and H28, 1,000; MeT-5A, 3,000 cells/well, respectively). After 12 days, the colonies were fixed with 2% paraformaldehyde, and stained with 0.1% crystal violet.

### Analysis of Annexin V binding and propidium iodide (PI) incorporation

Annexin V binding and PI incorporation were detected with a MEBCYTO apoptosis kit (MBL). The cells were harvested and washed with PBS, then stained with fluorescein isothiocyanate (FITC)-labeled Annexin V and PI. Fluorescence intensity was determined using a FACScalibur flow cytometer (BD Biosciences).

### Western blotting

Whole cell lysate was prepared by lysing the cells with extraction buffer [150 mM NaCl, 1% Triton X-100, 10 mM Tris-HCl (pH 7.5), 5 mM EDTA (pH 8.0), 1 mM phenylmethylsulfonyl fluoride (PMSF), 1 tablet/10 ml protease inhibitor cocktail (Roche Applied Science, Indianapolis, IN, USA)]. Soluble proteins were collected and concentrations were measured by DC protein assay kit (Bio-Rad, Richmond, CA, USA). Proteins were separated by SDS-PAGE and transferred to polyvinylidine difluoride (PVDF) membrane (GE Healthcare, Little Chalfont, UK). The membrane was blocked by 5% fat-free skim milk, then primary and secondary antibodies were added to the membrane, respectively. Bands of interest were detected using ECL Western blotting detection regents (GE Healthcare).

### Calculation of CI values

To assess whether the combination effect is synergistic or additive, CI analysis was performed. CI, a numerical value calculated as described in equation below provides a quantitative measure of the extent of drug interaction.

$$\text{CI}={\text{C}}_{\text{SBL},\text{X}}/{\text{IC}}_{\text{X},\text{SBL}}+{\text{C}}_{\text{TRAIL},\text{X}}/{\text{IC}}_{\text{x},\text{TRAIL}}$$

C_SBL,X_ and C_TRAIL, X_ are the concentrations of SBL and TRAIL used in combination to achieve x% drug effect. IC_X,SBL_ and IC_x, TRAIL_ are the concentrations for single agents to achieve the same effect. When the concentration of SBL was set to 0.5 *μ*M, treatment with TRAIL carried out in the range of 0.1–2.0 ng/ml, and when the concentration of TRAIL was set to 1 ng/ml, treatment with SBL carried out in the range of 0.1–2.0 *μ*M. CI of <, = and >1 indicates synergy, addictively and antagonism, respectively.

### Detection of the reduction of mitochondrial membrane potential (MMP)

MMP was assessed using a fluorescent probe 5,50,6,60-tetrachloro-1,10,3,30-tetraethyl-benzamidazolocarbocyanin iodide (JC-1, AnaSpec, Fremont, CA, USA). Cells were cultured in the conditions of each experiment and then incubated with JC-1 (2 *μ*M) dye diluted in culture medium at 37°C for 15 min. The cells were washed with PBS and analyzed using FACScalibur (Becton-Dickinson).

### Knock-down of expression of Bid by siRNA treatment

Introduction of siRNA into H28 cells was performed by lipofection method. Bid specific siRNA (10 *μ*M, sense; GGG AUGAGUGCAUCACAAATT, antisense; UUUGUGAUGC ACUCAUCCCTG) and Lipofectamine were mixed with Opti-MEM and added to the cells. After 4-h incubation, the medium was replaced with fresh medium and cells were cultured for 48 h. Thereafter, cells were harvested, plated in fresh dishes, and cultured for 24 h, and then siRNA was introduced again as above. The cells were used for the experiments 48 h from the final transfection.

### Statistical analysis

Results were collected from three independent experiments, each performed in triplicate, and data are expressed as the mean ± SD. Statistical analysis was performed using GraphPad Prism 3.0 and comparisons were performed using Student's t-test, one-way or two-way analysis of variance (ANOVA), followed by Bonferroni's *post hoc* tests.

## Results

### SBL shows anti-proliferative effects on malignant mesothelioma cells but not on non-malignant mesothelial cells

**Figure 1.**
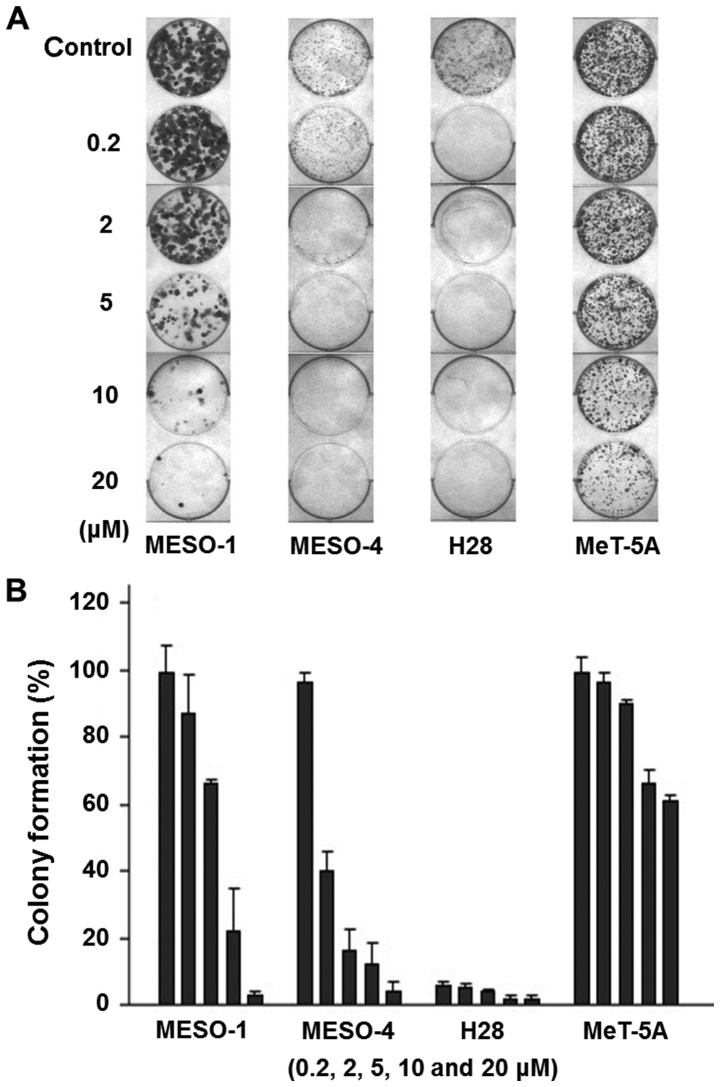
Effect of SBL on clonogenic potential of malignant mesothelioma cells and non-malignant mesothelial MeT-5A cells. (A) Cells were precultured for 24 h, then treated with increasing doses of SBL (0.2, 2, 5, 10 and 20 *μ*M) for 48 h. After treatment, cells were washed with medium and plated in a 6-well plate (MESO-1, 500; MESO-4 and H28, 1,000; MeT-5A, 3,000 cells/well, respectively). After 12 days, the colonies were fixed with 2% paraformaldehyde, and stained with 0.1% crystal violet. (B) Assays were done in triplicate, and the average of colony counts is presented.

Anti-proliferative effect of SBL to three malignant mesothelioma cell lines (H28, MESO-1 and MESO-4) and non-malignant mesothelial cells (Met-5A) was assessed by clonogenic assay. At the concentrations 5, 10 and 20 *μ*M of SBL, the colony formations of MESO-1 and MESO-4 cells were <70, 30 and 5% and 20, 15 and 5%, respectively. In H28 cells, the colony formation was <5% at all concentrations tested, while the colony formation of Met-5A cells were higher at 90, 65 and 60%, respectively (Fig. 1). H28 cells were the most sensitive in the cell lines tested, and sensitivity was observed in the order MESO-1, MESO-4 and Met-5A cells. These results indicate that SBL has selective anti-proliferative effects on malignant mesothelioma cells.

### SBL induces apoptosis to malignant mesothelioma cells but not to non-malignant mesothelial cells

**Figure 2.**
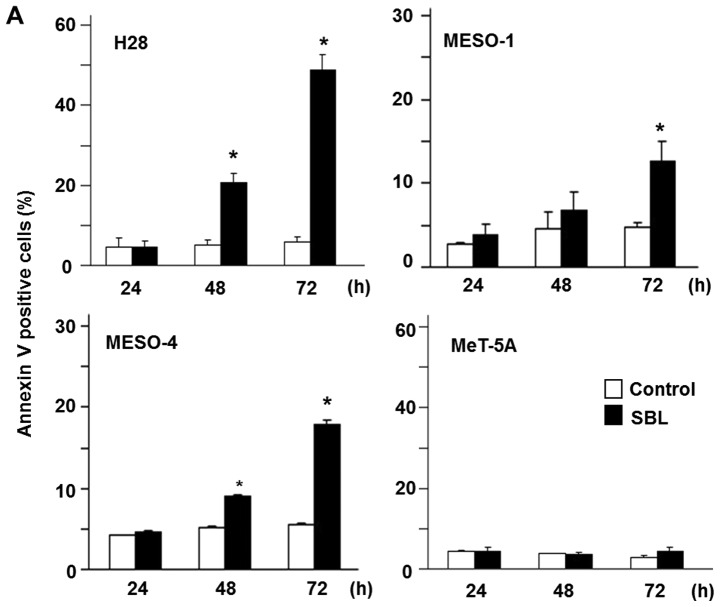

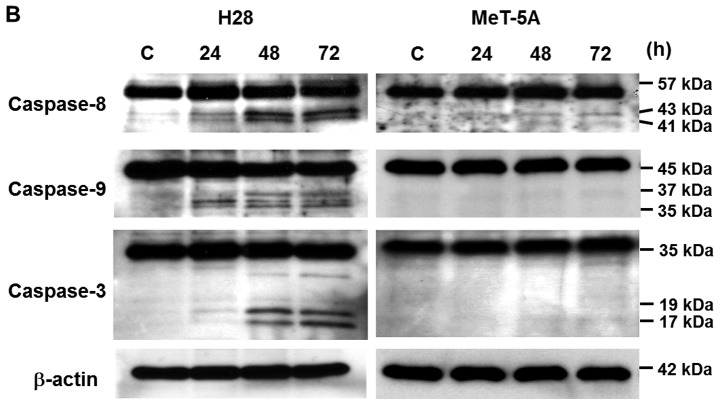

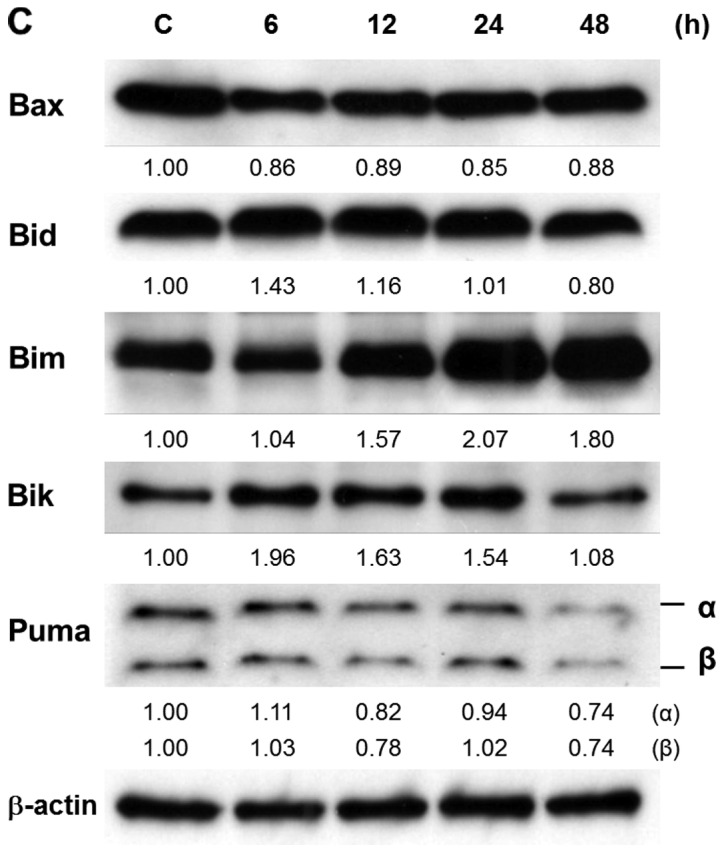

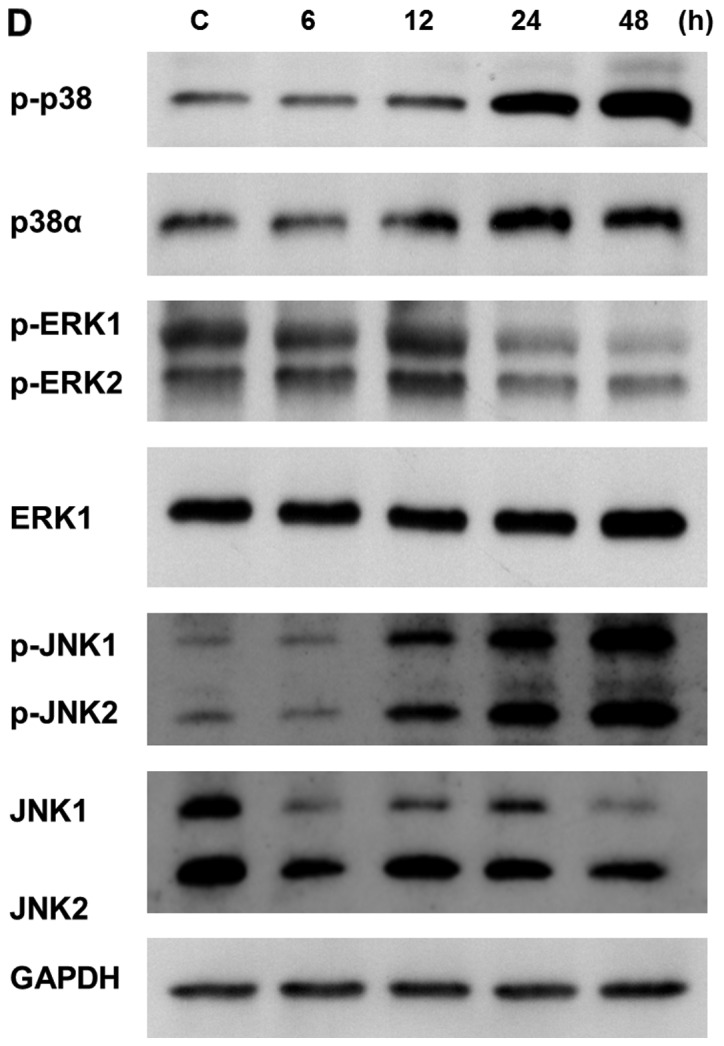
SBL induces apoptosis in malignant mesothelioma cells. (A) Binding of Annexin V in SBL-treated cells (H28, MESO-1, MESO-4 and MeT-5A). Cells were treated with SBL (5 *μ*M) for the indicated time. The percentage of Annexin V-positive cells was determined using flow cytometric analysis. Data are expressed as the mean ± SD of three independent experiments. ^*^P<0.05 versus control. (B) Caspase activation in SBL-treated H28 and MeT-5A cells. H28 and MeT-5A cells were treated with SBL (5 *μ*M) for the indicated time. The activation of caspase-8, -9 or -3 was determined by western blotting. β-actin was used as a standard to ensure equivalent loading of cell extracts. (C) Effect of SBL on expression of proapoptotic Bcl-2 family proteins. H28 cells were treated with SBL (5 *μ*M) for the indicated time. Expression of Bax and BH3-only proteins (Bid, Bim, Bik and Puma) was determined. Bands in the western blotting were quantified by densitometry and expressed as a ratio of intensity of bands to β-actin (respective bands/actin). (D) Phosphorylation pattern of MAPKs in SBL-treated H28 cells. H28 cells were treated with SBL (5 *μ*M) for the indicated time. Expressions of each protein were detected by western blotting. GAPDH was used as a standard to ensure equivalent loading of cell extracts.

Next, we assessed whether the anti-proliferative effect on SBL to malignant mesothelioma cells is the resultant of apoptosis or not. Annexin V binding was observed in all three malignant mesothelioma cells from 24-h treatment of SBL in a time-dependent mannner, while Annexin V binding was not detected in Met-5A cells (Fig. 2A). Then, we analyzed activation of caspases in SBL-treated H28 and Met-5A cells by western blotting. Apparent activation of caspase-8, -9 and -3 was observed from 48-h treatment with SBL in H28, but not in Met-5A cells (Fig. 2B). Furthermore, to clarify the other factors involved in SBL-induced apoptosis, we assessed the expression of proapoptotic Bcl-2 protein members by western blotting. There were no alteration in expression of Bax, Bid, and Puma, but elevated expression of Bim was observed, and maximal at 24 h (Fig. 2C). Transient elevation of Bik expression was also observed as early as 6-h treatment with SBL. We also analyzed if mitogen-activated protein kinases (MAPKs) were activated in the process of SBL-induced apoptosis. Elevation of total p38 expression was observed from 24-h treatment with SBL. Phosphorylation of p38 and JNK was detected from 24- and 12-h treatment with SBL, respectively, in a time-dependent manner. On the other hand, phosphorylation of ERK was diminished from 12-h treatment with SBL. These results indicate that SBL induces apoptosis selectively to malignant cells. Moreover, it was considered that Bcl-2 family proteins such as Bim and Bik, and p38 and JNK may be involved in apoptotic signal caused by SBL.

### Combinatorial treatment with SBL and TRAIL shows synergistic cytotoxity to H28 cells attributed to enhancement of apoptosis

**Figure 3.**
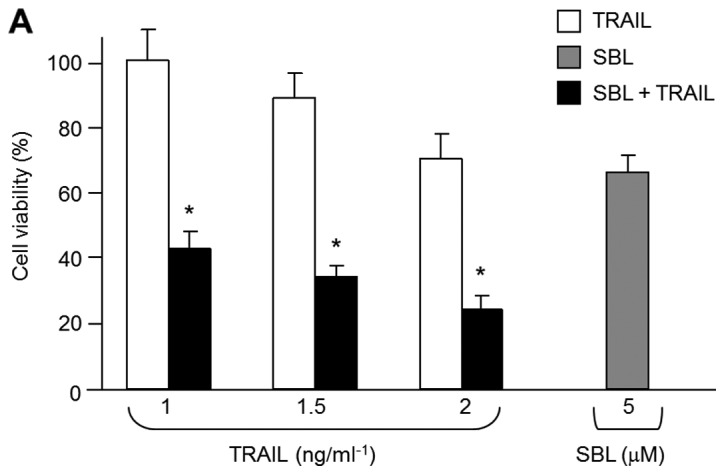

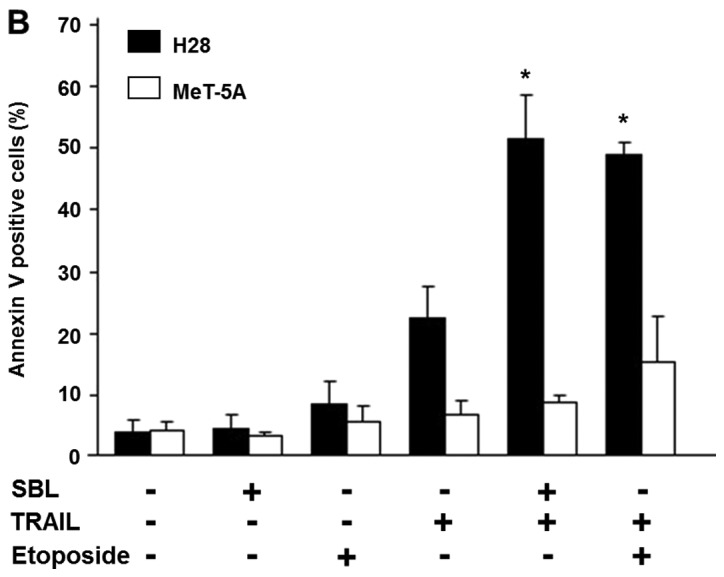

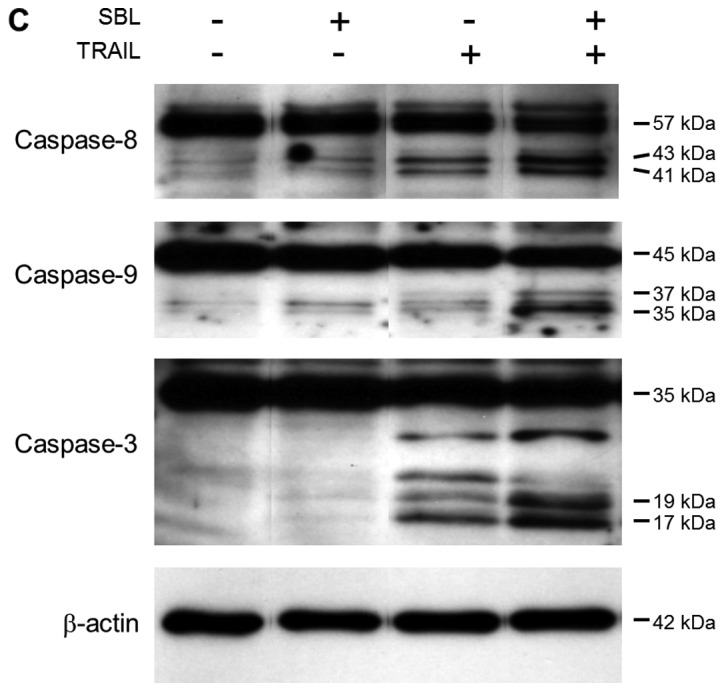
Combinatorial treatment with SBL and TRAIL shows synergistic cytotoxity. (A) Cytotoxic effect of combination of SBL and TRAIL in H28 cells. Cells were treated with SBL (5 *μ*M) and/or TRAIL (1, 1.5 and 2 ng/ml) in combination for 24 h. The viability was determined by WST-1 assay. Data are expressed as the mean ± SD from three independent experiments in triplicate. ^*^P<0.05 versus SBL alone. (B) Binding of Annexin V in combination-treated cells. H28 or MeT-5A cells were treated with SBL (5 *μ*M), etoposide (50 *μ*M) and/or TRAIL (2 ng/ml) for 24 h. The percentage of Annexin V-positive cells was determined using flow cytometric analysis. Data are expressed as the mean ± SD of three independent experiments. ^*^P<0.02 versus TRAIL alone. (C) Caspase activation in combination-treated H28 cells. Cells were treated with SBL (5 *μ*M) and/or TRAIL (2 ng/ml) for 24 h. The activation of caspase-8, -9 or -3 was determined by western blotting. β-actin was used as a standard to ensure equivalent loading of cell extracts.

Studies were designed to investigate the effectiveness of combinatorial treatment with SBL and other anticancer reagents. Fas ligand and TNFα reportedly kill not only tumor cells but also normal cells, whereas, TRAIL is promising because of its high selectively to cancer cells. We analyzed if the antitumor effects were enhanced by combinatorial treatment with SBL and TRAIL. Twenty-four-hour treatment of SBL (5 *μ*M) and TRAIL (2 ng/ml) alone resulted in loss of viability to 66.7 and 70.9%, respectively. Combinatorial treatment with SBL and TRAIL decreased the viability to 24.7% (Fig. 3A). When the concentration of SBL was set to 0.5 *μ*M or TRAIL was 1 ng/ml, the CI values were 0.63 and 0.68, respectively, which indicate synergistic effect (Table I). To study the mechanism of synergistic effect of SBL and TRAIL, we tested whether apoptosis is enhanced in combinatorial treatment. Etoposide that enhances TRAIL-induced apoptosis, was assessed together as a positive control. Similarly to combinatorial treatment with etoposide and TRAIL, Annexin V binding was significantly increased in combinatorial treatment with SBL and TRAIL in H28 cells, whereas these effects were not observed in Met-5A cells (Fig. 3B). Furthermore, activation of caspase-8, -9 and -3 was significantly enhanced in combinatorial treatment with SBL and TRAIL (Fig. 3C in H28 cells). These results suggest that synergistic cytotoxicity of SBL and TRAIL are caused by the enhancement of apoptosis.

### Synergistic effect of SBL and TRAIL is mediated by Bid

**Figure 4.**
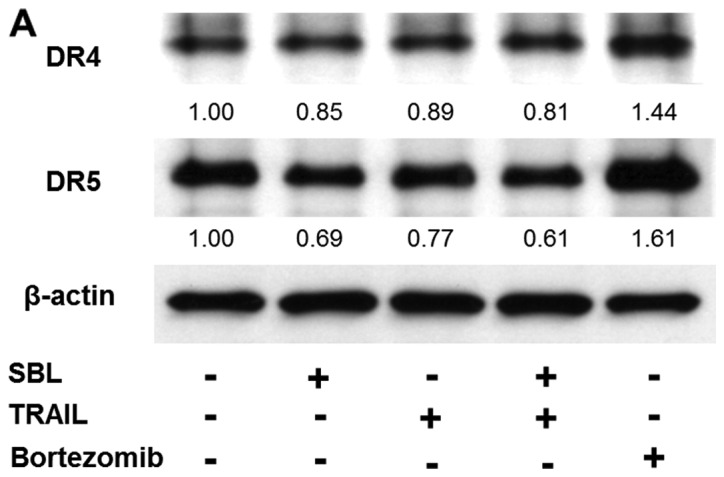

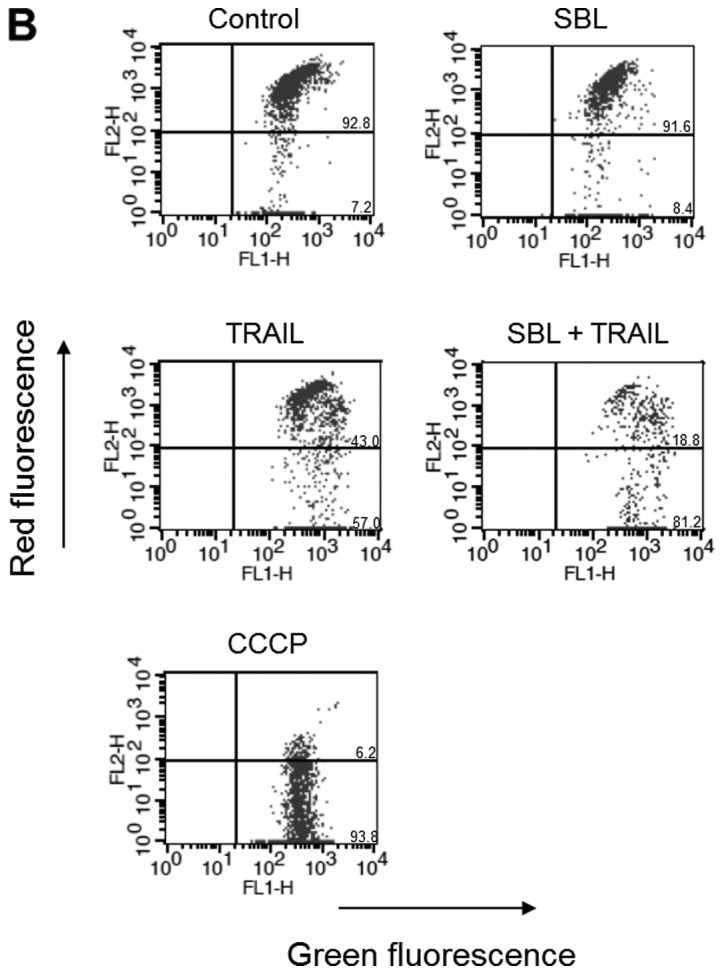

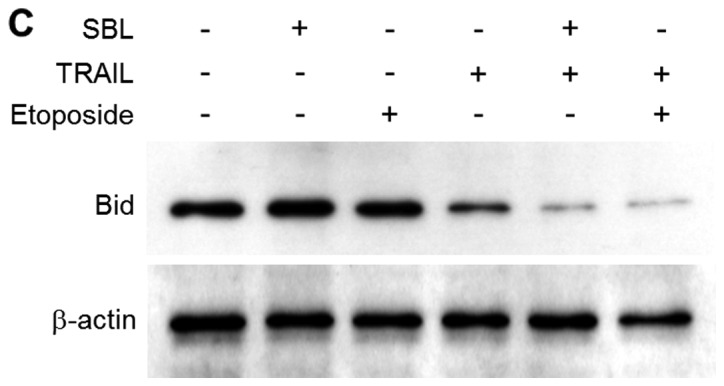

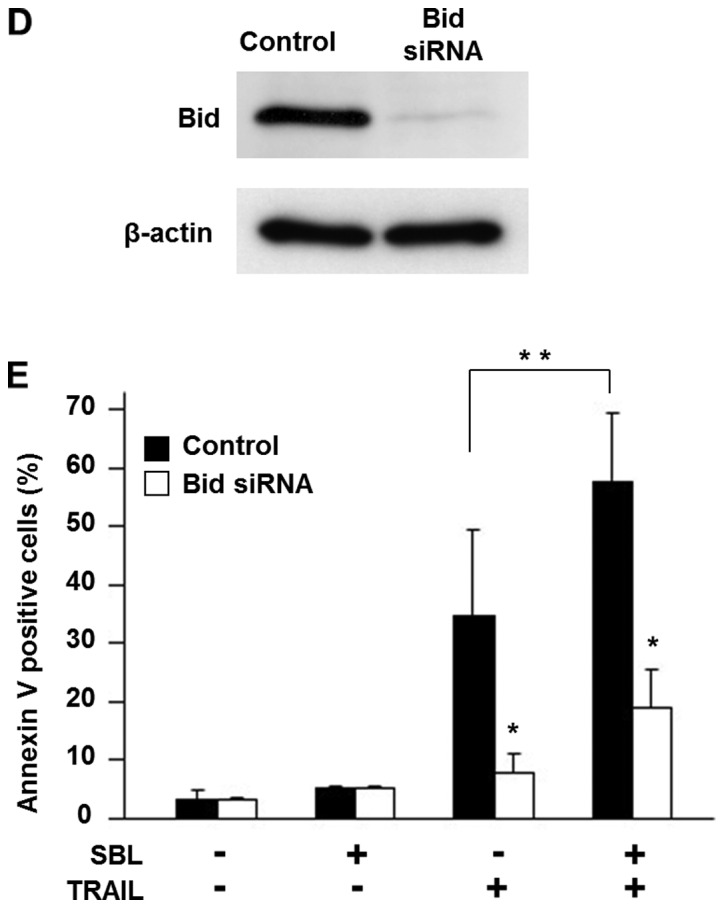

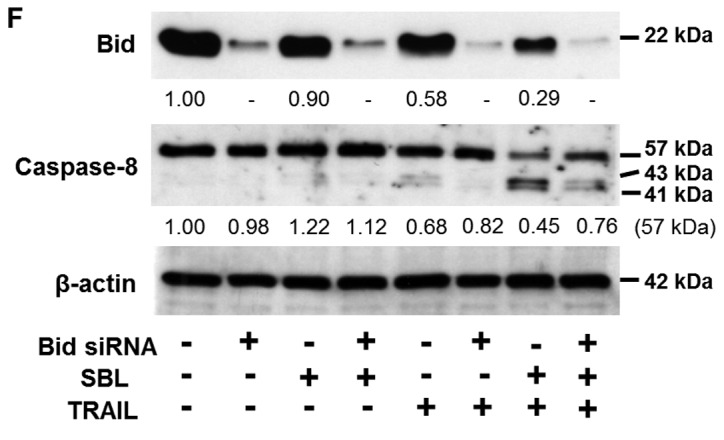
Mechanistic analysis of synergistic effect of SBL and TRAIL. (A) Effect of SBL on expression of DR4 and DR5. H28 cells were treated with SBL (5 *μ*M) and/or TRAIL (2 ng/ml) in combination for 24 h. Expression of DR4 and DR5 was determined western blotting. Bortezomib (10 nM), known to upregurate DR4 and DR5, was used as positive control. Bands in the western blotting were quantified by densitometry, and expressed as a ratio of intensity of bands to β-actin (respective bands/actin). (B) Change of MMP in combination-treated H28 cells. Cells were treated with SBL (5 *μ*M) and/ or TRAIL (2 ng/ml) for 24 h and then exposed to JC-1 for 30 min. Change of MMP was determined using flow cytometric analysis. The percentage of cells divided into lower right-hand (LR) quadrant and upper right-hand (UR) quadrant is indicated. (C) Bid-mediated synergistic effect in combinatorial treatment with SBL and TRAIL. Bid cleavage in combination-treated H28 cells. Cells were treated with SBL (5 *μ*M), etoposide (50 *μ*M) and/or TRAIL (2 ng/ml) for 24 h. The cleavage of Bid was determined by western blotting. β-actin was used as a standard to ensure equivalent loading of cell extracts. (D) RNA knock-down of Bid. H28 cells were subjected to two sequential rounds of transfection with Bid-specific siRNAs or vehicle (control). Bid expression is almost abrogated by the specific RNAi; there is no effect on expression of actin. (E) Effect of RNA knock-down of Bid on apoptotic facilitation between SBL and TRAIL in H28 cells. H28 cells subjected to two sequential rounds of Bid specific RNAi were treated with SBL (5 *μ*M) and/or TRAIL (2 ng/ml) for 24 h. Percentage of Annexin V-positive cells was determined using flow cytometric analysis. Data are expressed as the mean ± SD of three independent experiments. P<0.02 versus control transfected with vehicle (*) or TRAIL alone (**). (F) Effect of RNAi knock-down of Bid on caspase-8 cleavage facilitated by combination of SBL and TRAIL in H28 cells. H28 cells transfected with Bid-specific siRNAs or vehicle were treated with SBL (5 *μ*M) and/ or TRAIL (2 ng/ml) for 24 h. Bid expression and caspase-8 activation was detected by western blotting. Bands in the western blotting were quantified by densitometry, and expressed as the ratio of intensity of bands to β-actin (respective bands/actin).

Next, we analyzed how the synergistic effects were induced in combinatorial treatment with SBL and TRAIL. Some anticancer reagents increase the expression of DR4 and DR5 and shows synergistic antitumor effect with TRAIL. We analyzed the expression of DR4 and DR5 in SBL-treated H28 cells by western blotting to elucidate the possibility if SBL could increase the expression of these receptors. Bortezomib that increases the expression of DR4 and DR5 was used as a positive control. We detected an increase of DR4 and DR5 expression in bortezomib-treated cells, but there are no increments of DR4 and DR5 expression in SBL and/or TRAIL-treated cells (Fig. 4A). These results indicate that expression of DR4 and DR5 is not related to enhancement of apoptosis in combinatorial treatment with SBL and TRAIL.

Mitochondrial membrane depolarization is an indicator of apoptosis. We measured the MMP by using the mitochondrial membrane depolarization detector JC- 1. The mitochondrial membrane depolarization was significantly enhanced in combinatorial treatment with SBL and TRAIL, suggesting the involvement of mitochondrial perturbation in a synergistic mechanism (Fig. 4B).

Bid, a proapoptotic member of the Bcl-2 family, is cleaved by activated caspase-8, and then truncated Bid (tBid) translocates from the cytoplasm to the mitochondria, causing an efflux of cytochrome *c* from the mitochondria. We observed truncation of Bid in combinatorial treatment with SBL and TRAIL in H28 cells. Fig. 4C showed that tBid was significantly increased in combinatorial treatment with SBL and TRAIL, similarly to combinatorial treatment with etopside and TRAIL. Next, we assessed the contribution of enhanced Bid activation to synergistic effect of SBL and TRAIL by the knock-down of Bid. Enhancement of Annexin V binding in combinatorial treatment with SBL and TRAIL was significantly decreased by treatment of Bid siRNA (Fig. 4D and E). Furthermore, the enhanced activation of caspase-8 was also diminished by Bid siRNA (Fig. 4F). These results indicate that truncation of Bid is increased in combinatorial treatment with SBL and TRAIL and is plays an important role in synergistic apoptosis execution.

## Discussion

In this study, we showed that SBL inhibited cell growth of the various malignant mesothelioma cells, but not of the non-malignant mesothelial cells (Fig. 1). SBL-induced cytotoxity was accompanied by typical apoptotic changes, and these effects were only seen in malignant mesothelioma (Fig. 2). Thus, the new mechanistic and cancer selective properties of SBL can be assumed for the candidate for new kind of cancer therapy.

Combination therapy has been the standard of care, especially in cancer treatment, since it is a rational strategy to increase response and tolerability, and to decrease resistance. We studied here, the validity of the combinatorial treatment with SBL and TRAIL. TRAIL represents a promising candidate for the cancer therapy, because it causes apoptosis selectively to cancer cells (18,19). There are members of receptors for TRAIL including death receptors (DRs), DR4 and DR5, mediating induction of apoptosis, and the decoy receptors (DcR), DcR1 and DcR2 which fail to induce apoptosis (20–22). The selectivity of TRAIL to cancer cells is attributed to the fact that cancer cells have been shown to highly express the death receptors, whereas normal cells highly express the DcRs. However, many tumor cells are resistant to TRAIL in clinical trials. It is suggested that the low efficiency of TRAIL receptor agonists was due to the induced resistance (23–26). Efforts have been made to overcome the resistance and to improve the efficacy of TRAIL. There are reports that the apoptosis induced by TRAIL is enhanced by some other reagents (27,28). Bortezomib enhances the cytotoxity of TRAIL by increasing the expression of DR4 and DR5. We assessed the possibility that SBL is able to increase the expression of DR4 and DR5, and clarified that their expressions are not affected by SBL treatment (Fig. 4A). Engagement of death ligands to death receptors leads to activation of caspase-8, then activated caspase-8 transduces two different pathways dependent on cell types (29,30). In one pathway the activated caspase-8 directly activates effector caspases such as caspase-3 (31,32), in another pathway activated caspase-8 evokes mitochondrial perturbation through cleavage of Bid, and signal of apoptosis is amplified between caspase activation, mitochondria perturbation and Bid truncation (33). Cell types can be distinguished between type I cells using the former pathway (SKW6.4, H9) or type II cells using the latter pathway (Jurkat, CEM) (34). Etoposide was reported to sensitize malignant mesothelioma M28 cells to TRAIL-induced apoptosis, and this synergic effect requires amplification of death signals by cleavege of Bid, suggesting that M28 cells belong to type II cells (35). We investigated what the mediator of synergistic effect is between SBL and TRAIL. We found enhancement of apoptosis and mitochondrial depolarization in combination-treated H28 cells (Figs. 3 and 4B). Further study revealed increased cleavage of Bid, and it was important for amplification of apoptosis such as enhancement of caspase-8 activation in combination-treated cells (Fig. 4C–F). Our results also suggested that H28 cells belong to type II cells, because the cleavage of Bid enhances the apoptotic signals. Interestingly, Abayasiriwardana *et al* (36) reported that anisomycin, a translation inhibitor, lowers threshold for mitochondrial perturbation through Bim, and suggested that the contribution of JNK for the stability of Bim. Nikrad *et al* (37) and De Wilt *et al* (38) reported that bortezomib sensitizes cells to killing by TRAIL through not only the increment of expression of DR4 and DR5 but also the regulation of expression of Bcl-2 family including Bik and Bim. In the case of SBL, we detected elevated expression of Bik and Bim in SBL-treated cells. While anisomysin activated JNK and ERK but not p38 MAPKs (36), SBL activated JNK and p38 but not ERK (Fig. 2D). These observations suggest that Bcl-2 family proteins and MAPK signals play an important role in synergistic apoptotic cell death caused by combinatorial treatment.

**Figure 5.**
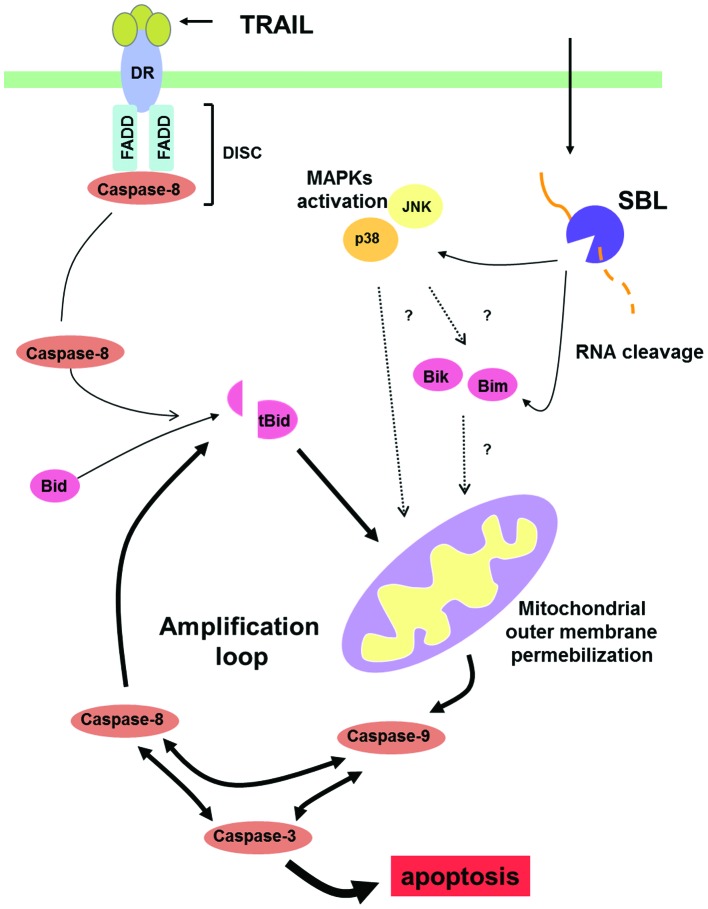
Proposed model for mechanism of apoptosis amplified by SBL and TRAIL in H28 cells. SBL catalyzes cleavage of cellular RNAs, and increases expression of Bik and Bim, phosphorylation of p38 and JNK MAPKs. On the other hand, TRAIL activates DR mediated by death-inducing signaling complex (DISC), which is formed by recruitment of Fas-associated death domain protein (FADD) and caspase-8. Caspase-8 activates Bid. These two signals induce apoptotic ‘amplification loop’ (thick line) associated with mitochondrial outer membrane permeabilization and caspase activation.

In conclusion, SBL induced selective apoptosis to malignant mesothelioma cells. The combinatorial treatment with SBL and TRAIL induced synergistic apoptosis to malignant mesothelioma. The predicted mechanism is shown in Fig. 5. The synergistic effects were caused through enhancement of Bid cleavage and caspase activation. Bcl-2 family proteins and MAPKs may be involved in the synergistic mechanism. Eventually, apoptotic signal is amplified by amplification loop consisted of caspase activation and mitochondria perturbation and truncation of Bid. The cytotoxic effects of SBL and/or TRAIL were not observed in non-malignant mesothelial cells. Therefore, our results suggest that the combination of SBL and TRAIL can be an effective treatment for malignant mesothelioma.

## Figures and Tables

**Table I.** Calculation for the combination of SBL and TRAIL.

Combination index (CI)	
SBL (0.5 *μ*M)	TRAIL (1 ng/ml)
0.63	0.68

CI values of >1 and <1 indicate drug antagonism and synergism, respectively.
